# Neuroinflammation: A Potential Risk for Dementia

**DOI:** 10.3390/ijms23020616

**Published:** 2022-01-06

**Authors:** Md Afroz Ahmad, Ozaifa Kareem, Mohammad Khushtar, Md Akbar, Md Rafiul Haque, Ashif Iqubal, Md Faheem Haider, Faheem Hyder Pottoo, Fatima S. Abdulla, Mahia B. Al-Haidar, Noora Alhajri

**Affiliations:** 1 Department of Pharmacology, Faculty of Pharmacy, Integral University, Lucknow 226021, India; ahmad.mdafroz@gmail.com (M.A.A.); mohdkhushtar@gmail.com (M.K.); fhaider89@gmail.com (M.F.H.); 2 Department of Pharmaceutical Sciences, University of Kashmir, Hazratbal, Srinagar 190006, India; ozaifa.scholar@kashmiruniversity.net; 3 Pharmaceutical Medicine, Department of Pharmacology, School of Pharmaceutical Education and Research, Jamia Hamdard, New Delhi 110062, India; akbarpharmas@gmail.com (M.A.); asifiqubal2013@gmail.com (A.I.); 4 Department of Pharmacognosy, School of Pharmacy, Al-Karim University, Katihar 854106, India; hrafiul@gmail.com; 5 Department of Pharmacology, College of Clinical Pharmacy, Imam Abdulrahman Bin Faisal University, Dammam 31441, Saudi Arabia; fhpottoo@iau.edu.sa; 6 College of Medicine and Health Science, Khalifa University, Abu Dhabi P.O. Box 127788, United Arab Emirates; 100058320@ku.ac.ae (F.S.A.); 100053510@ku.ac.ae (M.B.A.-H.); 7 Department of Medicine, Sheikh Shakhbout Medical City (SSMC), Abu Dhabi P.O. Box 127788, United Arab Emirates

**Keywords:** dementia, Alzheimer’s disease, neuroinflammation, phagocytosis, microglial cell activation, cytokines, chemokines

## Abstract

Dementia is a neurodegenerative condition that is considered a major factor contributing to cognitive decline that reduces independent function. Pathophysiological pathways are not well defined for neurodegenerative diseases such as dementia; however, published evidence has shown the role of numerous inflammatory processes in the brain contributing toward their pathology. Microglia of the central nervous system (CNS) are the principal components of the brain’s immune defence system and can detect harmful or external pathogens. When stimulated, the cells trigger neuroinflammatory responses by releasing proinflammatory chemokines, cytokines, reactive oxygen species, and nitrogen species in order to preserve the cell’s microenvironment. These proinflammatory markers include cytokines such as IL-1, IL-6, and TNFα chemokines such as CCR3 and CCL2 and CCR5. Microglial cells may produce a prolonged inflammatory response that, in some circumstances, is indicated in the promotion of neurodegenerative diseases. The present review is focused on the involvement of microglial cell activation throughout neurodegenerative conditions and the link between neuroinflammatory processes and dementia.

## 1. Introduction

There are more than 703 million people aged above 65 years, worldwide, a number that is predicted to reach 1.5 billion by 2050 [1,2]. Globally, the dramatic increase in life expectancy is followed by a rise in age-related illness. Older people are expected to spend more of their later lives in poor health with a decreased quality of life [3]. Dementia is a leading factor responsible for dependency and mental disability among more than 50 million elderly people world-wide [4].

Dementia has the highest economic cost of adult neurological disorders and is the second most common factor responsible for mortality due to neurological disease after cerebrovascular disease. The average costs of the management and treatment of dementia are estimated to be betweenUSD600 billion and USD 1 trillion. Therefore, finding the right way to avoid the expansion of the disease is crucial [5]. Dementia is a syndrome that causes persistent decrease in cognitive and behavioral function of the brain that hampers the ability to perform day-to-day activities [6,7]. Dementia is associated with a large group of neurological pathologies such as frontotemporal dementia (FTD), Parkinson’s disease (PD), Alzheimer’s disease (AD) and Lewy body dementia (LBD) [7,8]. The earliest form of neurological disorders, whose manifestations are predominantly cognitive, is termed as primary dementia (Table 1) [6]. Another early sign of dementia, known as mild cognitive impairment (MCI), is a state between aging and dementia, with reduced cognitive ability and retained self-supporting function. Dementia that progresses from normal cognition to severe dementia in less than two years is characterized as “rapidly progressive dementias” (RPDs) [9,10]. Dementia is always latent and hard to detect in the initial phase, making it a double challenge for primary care professionals. Initial detection is important as this enables the patients and their families to participate in a supportive programmes, strategize their future and reduce the major psychological suffering experienced by patients. Information on psycho-social and the availability of medical assistance would increase the morale of patients [5,11,12,13]. The early identification and interference of dementia may postpone admission to the nursing home, and thus reduce the huge socio-economic expenses [14].

Within the last quarter of the 20th century, dementia appears to be one of the most under-diagnosed conditions in primary assessment. Dementia is primarily diagnosed on the basis of symptoms, as well as the caregiver’s concerns or obvious memory impairment [15,16]. There has been substantial educational work on primary care dementia, supported by charitable organizations, and focused initiatives. The Audit Commission has emphasized the strategic needs and thoughtful methods for identifying and controlling dementia [17]. The National Service Framework for Older People has agreed to promote the progress of common practices in dementia care through designed protocols for screening and management of dementia [18,19,20,21].

Until now, no specific treatment is available for dementia or AD [16], but various therapeutic approaches such as amyloid-driven immunotherapy, inhibition of amyloid synthesis, and extracellular tau immunotherapy focus on alleviating symptoms [22,23,24]. Anti-amyloid beta monoclonal antibody was assumed as a promising agent for the management and treatment of AD, but when investigated for clinical efficacy, the monoclonal antibody solanezumab did not substantially impact cognitive decline [25].

Pharmacological approaches for the symptomatic management of dementia or AD are based on the modulation of various neurotransmitters such as acetylcholine, serotonin, and norepinephrine [26,27,28]. Non-pharmacological interventions designed to curb excessive disability consist of cognitive therapy, lifestyle changes, and patient education [18,29,30].

Neuroinflammation generally plays a role in reduced cognitive function, and this can turn pathological, such as in dementia associated with old age, and AD [8,31]. While there is no clear cause of AD, it has been found that chronic inflammation can lead to the production of Aβ plaques, which lead to further aggregation and inflammation, impacting neuron synapses. Aβ is a major trigger of neurodegeneration in AD, as it initiates a proinflammatory response [32]. This dysregulated inflammatory response impacts long-term brain function and furthers neurodegeneration. Examples of proinflammatory factors released in this immune response include cytokines (IL-1β, IL-6, IL-18, TNF-α,), chemokines (CCL2, CCL3, CXCL8), transcription factors (NF-κB), and peptides (bradykinin) [33,34,35,36]. However, it is the lack of the resolution of this response that leads to a pathology. Older brains maintain a level of chronic inflammation that risk losing the ability to clear the infection, as there is desensitization [32,37]. The blood–brain barrier also gets leaky as the brain ages, where unresolved inflammatory cells can cross. Microglia in an aged brain also tend to release more inflammatory cytokines, mainly IL-1β, IL-6 and TNF-α [38,39]. Chronic activation of microglia and inflammatory responses leads to the increased presence of tau by activating kinases that phosphorylate the protein, with TNF-α leading to increased aggregation in neurons when tested in vitro [40] This review aims to investigate the role of neuroinflammation in the development of dementia including the involved markers, modulators and risk factors.

## 2. Diagnosis of Cognitive Decline

Age-related cognitive changes are not to be generalized, as they are quite variable. The differentiation of dementia from normal age-related cognitive decline is difficult. The manifestation of dementia can vary from mild loss of cognition to complete loss of memory [41]. The greatest risk factor for cognitive decline is age, which in itself increases physiological inflammation. Inflammation has been linked to an increased risk for cognitive decline, contributing to the risk of dementia [42]. However, there is a line between normal and abnormal cognitive decline, which can be difficult to distinguish, but it is important to do so, as it has implications on the acquisition of pathologies [8].

Cognitive aging depends on multiple factors, with a model suggesting that it is mainly due to the decline of the immune system (immunosenescence), vascular aging (vascular inflammation), and brain aging (neuroinflammation). These factors result from life-long insults to the brain, including exposure to antigens, and all involve inflammation of some sort [43].

### 2.1. Cognitive Decline

Cognitive decline appropriate for increased age has varied manifestations. While a patient or their partner may report deterioration in cognitive ability and performance at work along with anxiety, the patient needs to be referred only after a primary care doctor raises concerns regarding background awareness or cognitive screening [28,44].

Neuronal death is a result of neurodegenerative disease. Etymologically, the term is derived from the prefix “neuro-,” which refers to nerve cells (i.e., neurons) and, “degeneration”, which refers to a phase of deteriorating structure or function in tissues or organs [45]. Neurodegenerative implies that neurons eventually stop working or start functioning poorly and inevitably die. This synaptic disconnection can lead to several malfunctions. Neurodegeneration refers to a neuron’s gradual atrophy and dysfunction, which is found in AD [28,46]. Neurodegeneration has become a widely used term with a generally known meaning, and in a strict sense of the term, refers to any neurological disorder primarily affecting neurons. In general, neurodegenerative diseases constitute a broad community of neurological diseases with heterogeneous clinical and pathological manifestations that affect specific subsets of neurons in various functional anatomical structures. They emerge relentlessly for unclear causes and developments. Increased age is the most consistent risk factor for developing a neurodegenerative disease, especially AD or PD [47,48].

Over the past century the growth rate of the population aged 65 and over in developed countries has significantly surpassed that of the population as a whole. It can also be expected that the proportion of elderly people will double in few years. This may be reflected in the proportion of those afflicted with neurodegenerative diseases, and thus would be a source of great concern on various levels such as mental, physical, and financial pressures on patients, as well as on policymakers [45]. The problem is exacerbated by the fact that some approved medications for several neurodegenerative diseases can alleviate symptoms to a certain extent but are associated with debilitating side effects in chronic users, with no appearance of halting degenerative advancement.

### 2.2. Aetiology of Neurodegenerative Disorders

The limitations of our understanding of the causes and mechanisms by which neurons die in neurodegenerative diseases have impeded the development of successful preventive or protective therapies [47]. Selected genetic and molecular developments related to the biology of neurodegeneration, such as apoptosis, oxidative stress, and mitochondrial dysfunction, have been analyzed in this perspective sequence [49]. The causes of neurodegenerative diseases are largely unknown, with a few exceptions. Even if they have been established, the mechanisms by which the disease is triggered remain speculative at best. One of the most heated debates on the etiology of neurodegenerative disorders is on the relative roles of genetic and environmental causative factors in disease initiation [50]. There is substantial family incidence of certain neurodegenerative diseases, indicating a genetic basis. The disease acts as an autosomal dominant trait among these affected families. Others are essentially sporadic in addition to these “pure” hereditary neurodegenerative disorders. However, they show a small group of patients in whom the disorder is inherited. Instead, it seems that harmful environmental factors may be primary suspects in triggering neurodegenerative processes [51].

### 2.3. Cognitive Downturn

Impairment of cognitive functions is the primary characteristic of dementia, which often manifests as memory deficits and a decline in cognitive skills [51]. Neuropsychological tests are the gold standard for cognitive testing, where an individual’s cognitive performance is expressed as the standard deviation (SD) from the mean performance of a cohort of cognitively stable individuals [6].

### 2.4. Chronic Decrease in Functionality

The regular activities of any individual are divided into two sets of activities—basic activities of daily living (BADL) and instrumental activities of daily living (IADL) [52]. BADL relate to fundamental tasks of self-care like feeding, bathing, using the toilet, and wearing clothes. At the same time, IADL comprises more complicated tasks, such as managing medicines, finances, transportation, meal preparation, and shopping. A chronic decrease in functionality refers to the inability of a person to execute IADL, with later stages of dementia affecting BADL [7,52].

### 2.5. Mild Cognitive Impairment

MCI is a transitional condition between normal cognition and dementia, essentially with retained mental ability. In older age, cognitive capacity is generally assumed to decline. The age-related decreases in mental abilities are highly variable between different individuals and an average decrease can be seen across the population. Dementia is a condition with an underlying physical disorder, consisting of brain tissue loss, which can be seen by a brain scan or autopsy after death [53]. Dementia is incremental, with damage and symptoms getting worse over time. Therefore, it is not a natural product of aging but a consequence of an illness [54,55].

Damage to the brain is likely to begin decades before physical signs begin to appear. This very long latent period has made it very difficult to find a cure for dementia, as brain deterioration advances too far before the signs are evident [33,50]. MCI was initially thought of as a working phase of dementia, however, some studies have suggested that MCI mostly develops into full-blown dementia while a subset of the population returns to normal cognition after a while [3,56,57]. MCI is increasingly identified as a prelude to dementia but not as an early phase of dementia. Several phenotypes of MCI, such as those affecting episodic memory, seem to pose a greater chance of developing AD [18,58,59].

## 3. Types of Dementias

### 3.1. Primary Dementias

Primary dementia initially manifests as a mild cognitive decline and sometimes presents with neurodegenerative proteinopathies. In addition, some protein mismatching may result in neurodegeneration, neuroinflammation, and degeneration of glial cells. Primary dementia comprises of AD, diseases of the prions, and frontotemporal dementia [60]. Lewy body dementia (LBD) and Parkinson’s disease dementia (PDD) are proteinopathies that are characterized by extrapyramidal motor symptoms and are included in primary dementia because these are of the main reasons that cause dementia. Vascular dementia is not caused by protein abnormalities but presents as the second major trigger of dementia among older adults [18,60,61]. Primary dementia can be classified by its clinical characteristics, neuropathological phenotype, and neuropsychology. Different proteins aggregate to characterize various protein pathologies. For example, AD is caused by a dual proteinopathy linked to amyloid β and hyperphosphorylated tau proteins. Tau is also involved in a variety of other pathologies such as progressive supranuclear palsy (PSP), corticobasal degeneration (CBD) and Pick’s disease, [62,63].

### 3.2. Rapidly Progressive Dementia

Rapidly progressive dementias (RPDs) are neurological disorders that develop sub-acutely from normal cognition to dementia in one to two years. These can also present with characteristics other than cognitive impairment, such as extrapyramidal symptoms, behavioral problems, electroencephalogram changes, and myoclonus. The term was initially used for types of prion illness and their differential detection. Diagnostically, RPDs can be classified into three categories: prion diseases, atypical presence of other primary dementias, and autoimmune encephalitis [10]. The relative occurrence of each type depends upon the setting. While prion diseases are more prevalent in tertiary reference centers, encephalopathies are more likely to be investigated as RPD in a community setting. Primary dementias that progress to RPDs are mainly reported in the younger patients and can lead to changes in white matter. The presence of amyloid pathology in positron emission tomography (PET) or cerebrospinal fluid (CSF) imaging may aid in the diagnosis, particularly among younger people. A comprehensive history, proof of neuroleptic responsiveness and, in some cases, examination of dopamine transporters can help identify the Lewy body disease. Fewer common causes of RPDs are secondary encephalopathies; diffuse neoplasms, herpes encephalitis, syphilis, Whipple’s disease, and infections such as the human immunodeficiency virus. Furthermore, temporal lobe epilepsy and non-convulsive seizures that are difficult to detect, particularly with epileptic status, may also resemble RPDs [10,64].

## 4. Risk Factors for Dementia

Non-adjustable risk factors responsible for dementia include age, genetic polymorphism, gender, family history, and race [65,66,67,68]. Aging is related to several brain changes that include hippocampus atrophy, an imbalance between amyloid-β synthesis and breakdown and the initiation of inflammation [69]. While the low levels of estrogen and other hormones after menopause are suggested to be the cause behind the increased prevalence of dementia in women compared to men [70]. Mutations in the genes of amyloid precursor protein (*APP*), presenilin 1 (*PSEN1*) and presenilin 2 (*PSEN2*) are associated with early AD. In contrast, polymorphism in the apolipoprotein E gene (*APOE*) is related to the late form of the disease [67,71,72].

Various published evidence demonstrates a link between the progress of cognitive injury and dementia with learning achievement and risk factors associated with lifestyle such as physical inactivity, an unhealthy diet, tobacco use, and detrimental alcohol use. Researchers have identified several risk factors that affect the likelihood of developing one or more kinds of dementia. Some of these factors are modifiable, while others are not. [73,74]. According to Kirsty B. et al., (2019), obesity is linked to higher long-term dementia incidence in those aged 65–74 years old [75]. Furthermore, a prospective analysis in 2019 showed that worse cognitive consequences were related to longer duration of diabetes duration and poor glycemic control over a 5-year follow-up period [76]. Another study illustrated that there was an increased risk of AD in widowed individuals compared to married ones [77].

Additionally, studies have reported aging as an important risk factor as various progressive neurological disorders such as AD, PD, neuroinflammation, etc., are positively correlated with aging [78]. The genetic factor is another important risk factor associated with dementia [79]. In the case of dementia associated with fatal familial insomnia, Creutzfeldt–Jakob disease and Gerstmann–Sträussler–Scheinker syndrome, patients with prion protein gene mutation are at higher risk [79]. Genetic mutation-related dementia has also been reported in FTDP-17 and Huntington’s disease [79].

Moreover, smoking and alcohol consumption have also been related to dementia and cognitive dysfunction [78]. Clinical studies have also shown that consumption of alcohol is associated with increased prevalence of dementia and other vascular diseases [80].

The association of metabolic syndrome and dementia, atherosclerosis and diabetes have extensively been studied [81]. Diabetes is directly associated with AD, stroke and cognitive dysfunction [81]. Atherosclerosis, which is characterized by increased deposition of LDL, has directly been reported with dementia [81]. In atherosclerosis, deposition of LDL interferes with the blood supply in the brain and manifest into dementia [73,74].

Modifiable risk factors include cognitive inactivity and social isolation. The risk factors incorporated in these guidelines have been selected based on recently published studies and guidelines, including the National Health and Care Excellence Institute (NICE, 2015); the World Alzheimer Report 2014 [27,82,83,84]. The cognitive impairment prevalence was higher in those who lived in rural areas compared to those who lived in urban areas. In addition, low income, less social participation, depression and disability appeared to be independent risk factors for the disease [85].

## 5. Neuroinflammation in Dementia

Inflammation is the body’s typical reaction to damage and stress; this includes redness and swelling associated with injury or infection. However, inflammation in the brain, known as neuroinflammation, has been identified and related to many conditions, including depression and multiple sclerosis [42,86,87]. The risk of AD has also recently been linked to neuroinflammation as well. Recently published reports have shown the involvement of proinflammatory markers in the brain as a more significant major risk factor for dementia than previously assumed [42]. The formation of reactive oxygen species (ROS), chemokines, cytokines and various second messengers mediate this inflammation. Resident peripherally derived immune cells, CNS glial cells such as microglia, astrocytes, and endothelial cells contain these mediators. These neuroinflammatory responses have immune, physiological, biochemical, and psychological effects. In addition, the course of primary stimulus significantly determines the extent of neuroinflammation. Inflammation can, for example, lead to immune cell recruitment, fluid retention, tissue injury, and eventually cell death [19,47,50]. Numerous studies have shown a connection between inflammation (an immunogenic response to pathogens, oxidative stress or irritants, injury) with cognitive decline, and a subsequent chance of dementia [20,88,89]. Inflammation is normally a defensive mechanism that helps in the curative process.

Nevertheless, sustained inflammation may damage the tissue. Modified inflammatory reactions to the immune system are adaptive and essential for properly stabilizing and recovering cells and tissues from insults like trauma, irritants, and pathogens [8,66,90]. According to various studies, a list of important risk factors for neuroinflammation such as aging, genetic factors, toxic metabolites, autoimmunity, microbial infections, traumatic brain injury, and air pollution was observed [88,90,91]. Normal aging is linked to increased and sustained inflammation throughout the body. Neurodegeneration, atherosclerotic processes, disabled neurogenesis, and persistent diseases are associated with ongoing increased levels of inflammation [90,91,92,93]. Although age-related inflammation may be a common result of immune senescence, it may also increase the susceptibility and threat of successive pathogenesis [93].

Neurological inflammation is a sensitive reaction of the CNS to noxious stimuli such as toxins, trauma, injury, ischemia, neoplasms, infections, etc., that impede homeostasis and lead to progressive neuronal death and loss of cognitive and motor functions in the CNS. The clinical evidence suggests that inflammation is a pivotal pathogenetic factor involved in neurodegenerative disorders and the interaction between the immune system and the nervous system [49]. The wide variety of cellular mechanisms, probably the same in aging and chronic disorders such as obesity, diabetes, dementia, etc., are considered to be the most pivotal in the generation of neuroinflammation [94,95,96]. Various research results on the inflammation seen in various parts of the brain of dementia patients are listed in Table 2.

**Figure 1:**
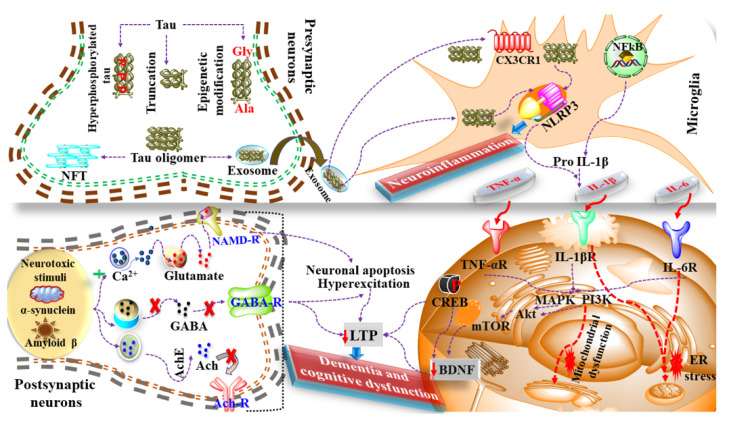
Role of neuroinflammation and dementia. The presence of coexisting neurological disorders such as Alzheimer’s disease, Parkinson’s disease, and exposure to neurotoxic stimulus causes increased chemokines and NLRP3 activation that eventually leads to neuroinflammation. Moreover, neurotoxic stimulus also induces neuroinflammation via modulation of MAPKs, Akt/PI3K, mTOR, ER stress and mitochondrial dysfunction. Additionally, these neurological disorders cause a dysfunctional release of neurotransmitters that further interferes with the function of CREB, BDNF, LTP, etc., and causes dementia.

Preclinical models and human research suggest that microglia, a part of the innate brain immune system, is triggered by dementia and various other neurodegenerative disorders. In addition, interventional genetic studies have shown a correlation between cognitive disease and gene-related mutations or polymorphisms in the immunogenic response. The most prevalent genetic risk factor, apolipoprotein E (*ApoE*), is a polymorphic lipoprotein that regulates the transportation as well as distribution of lipids such as cholesterol and others via *ApoE* receptors. There are three isoforms of human *ApoE* in which polymorphisms are present in the receptor-binding domain, *ApoE4* (R158, R112), E3 (R158, C112), and E2 (C158, C112), with the highest risk of AD conferred by *ApoE4*. Microglia and astrocyt-mediated inflammatory processes in the brain are dysregulated in an isoform-specific way, with *ApoE4* exhibiting the neuroinflammatory effect [103]. It has been reported that the *ApoE4* intensifies the neuroinflammatory innate immune response mediated by Aβ. Aβ mediates in response to systemic studies in mice and humans, which eventually manifests into neuronal dysfunction as shown in Figure 1 [32].

Considering the mechanism of dementia in AD and neuroinflammation, the tau protein undergoes hyperphosphorylation, epigenetic modification and truncation that cumulatively leads to the formation of NFT and the release of exosomes that further regulate the expression of chemokines such as CXCL3 and activation of NLRP3. Activated NLRP3 further manifests in the production of IL-1β that binds with the IL-1β receptor and imitates the cascade of neuroinflammation and dementia, as shown in Figure 1.

Additionally, Aβ, α-synuclein and neurotoxic stimulus are also involved in the mechanism of dementia via altering the release of various neurotransmitters, as shown in Figure 1. Therefore, on the one hand, the mediators of inflammation such as Il-1β, TNF-α, MAPKs cause alterations in the levels of neurochemicals such as CREB and BDNF while on the other hand, the discrete release of neurotransmitters causes alteration in long term potentiation (LTP) leading to neuroinflammation and dementia. Brain-derived neurotrophic factor (BDNF) is a neurotrophinthat is often associated with neurodegenerative disorders when low in level [104]. BDNF is typically associated with neuronal survival, synaptic formation, plasticity, and the alteration of inhibitory and excitatory mechanisms.

The presence of neurotoxic stimulus and coexisting neurological disorders causes an alteration in the level of BDNF and manifests into dementia and cognitive dysfunction.

Mutations in microglia-related genes such as SOD1 (superoxide dismutase 1), C9Orf72 (ORF 72 on chromosome 9), TBK1 (TANK-binding kinase 1), OPTN (optineurin), SQSTM1 (Sequesterome-1), and VCP (Valosin-containing protein), which activate microgliaincrease the production of neurotoxic factor production, leading to neuroinflammation. Mutations in genes such as C9Orf72, PFN1 (Profilin), TREM2 and GRN (granulin) have been shown to lead to phagocytosis and associated degradation pathways that are regulated by microglial cells [105]. Further damage to motor neurons in ALS is caused by changes in these processes, leading to neurodegeneration, encouraging the progression of the disease [106].

The inflammatory markers associated with neuronal damage include cytokines, transforming growth factor-beta (TGF-β), and interleukin-1β, causing direct synaptic microglial injury [65,107,108]. These worsen the neuronal network communication, synaptic impulse, thus accelerating synaptic failure and neurodegeneration. These findings offer the potential for immunotherapy action to stop or slow the development of dementia [56,109,110].

Different therapeutic strategies of AD target the neuroinflammation, including the inhibition of the gene expression of the cytokines and blocking their receptors, counteracting IL1-β and TNF-α effects by using antibodies that also bind their receptors and lowering the anti-inflammatory molecules Aβ and tau pathologies by limiting glial cell release of pro-inflammatory cytokines such as minocycline [111]. Urolithin A and actinonin can reduce tau and Aβ by increasing phagocytosis by enhancing the process of mitophagy which is the process related to getting rid of the damaged mitochondria [112]. On the other hand, neuroprotection could be achieved through polyphenolic compounds such as vitamin E that lower the production of Tumor necrosis factor-α and nitric oxide [113].

Furthermore, clinical trials that aim to study the efficacy of different drugs in the treatment of AD, specifically through working on the neuroinflammation process are in different phases. Indomethacin, a non-steroidal anti-inflammatory drug, is in the third phase of the clinical trial [114]. The drug activates peroxisome proliferator-activated receptor-gamma (PPAR-γgamma), a prototypical ligand-activated nuclear receptor. This activation reduces the activity of β-site amyloid precursor protein cleaving enzyme 1, which results in less Aβ production [115]. Another drug that is in the second phase of the clinical trials is Simufilam. It can decrease the inflammation and tau phosphorylation in animal models [116], while candesartan decreases the microglia hyperactivation in AD mice hippocampal region [117]. Minocycline has neuroprotective effects by reducing the levels of the inflammatory markers such as IL6, as well as the microglial activation [118]. On the other hand, Pioglitazone affects the process of phagocytosis which aids in getting rid of the deposited Aβ. It also decreases the interleukin-1β inflammatory cytokines production [119].

## 6. Mediators and Neuroinflammatory Modulators

Neuroinflammation is largely considered a pathological hallmark of neurodegenerative diseases. Neuroinflammation encompasses the multiple physiological responses elicited by microglial and astrocytic cells of the innate immune system and a plethora of proinflammatory factors that affect the environment of the CNS. It is largely triggered by potential toxins and infections that damage the cells and change neuronal activity. The primary responsibility of the CNS toward insult or injury involves the activation of microglia. However, chronic inflammation of neurons and sustained microglial activation results in significant proinflammatory cytokines and cell-damaging factors such as C-reactive proteins, chemokines, cytokines, etc., thus increasing local inflammation [35]. Other inflammatory modulators involved in neuroinflammation include caspases, prostanoids, neuroprotectin D, nitric oxide, reactive oxygen species, etc. [91,120,121]. Microglia are the phagocytes of the CNS and are present in the brain tissue. When microglial cells are activated in response to pathogenic stimulus, cascades for the production of various proinflammatory cytokines and neuroinflammatory events begin, and eventually lead to neuroinflammation [122]. Once microglial cells are activated, the accumulation of reactive astrocytes begins at the site of injury, similar to senile plaques in AD, but do not leave their site of origin and do not lead to scar formation [123]. Cytokines are the main contributors of neuroinflammation, including pro- and anti-inflammatory processes, chemotaxis, and neuronal injury [124]. Studies have also shown that microglial migration is regulated by chemokines at the sites of neuroinflammation, thus accentuating localized inflammation [125]. Studies have suggested that chronic inflammation within the central nervous system plays an important role in disease pathogenesis. The presence of microglial cells, and macrophages in CNS, are regulated in their normal physiological state and relate to injury and repair. Microglia can multiply whenever the brain is exposed to a noxious neurotoxic stimulus, a process known as priming. Priming allows microglia to be responsive to various secondary inflammatory stimuli, which can cause an excessive inflammatory response, especially if chronic, which can lead to neural damage. Neuronal aging is followed by an imbalance between anti-inflammatory and proinflammatory networks, increased development of reactive oxygen species, and increased phagocytic ability to drive microglial priming.

**Figure 2:**
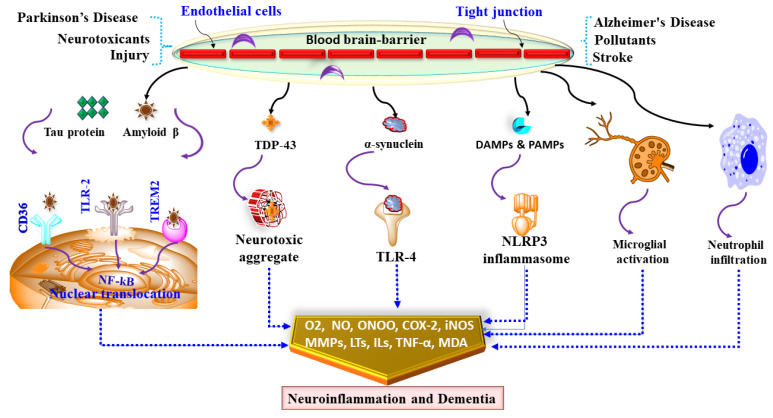
Showing mediators and neuroinflammatory modulators and dementia. Coexisting neurological disorders such as Alzheimer’s disease, Parkinson’s disease and exposure to neurotoxic stimulus cause damage to BBB via alteration in endothelial cells and tight junctions. Damaged BBB causes NLRP3, TLR-4 and microglial activation, macrophage infiltration and nuclear translocation of NF-kB that cumulatively causes oxidative stress and neuroinflammation that leads to dementia.

Systemic inflammatory diseases are also a major contributor to priming, and the hypothesis that neuroinflammation leads to cognitive impairment and dementia in T2D is primarily based on this idea. Studies suggest that chronic neuroinflammation has a more pivotal role than traditionally thought within AD pathogenesis. This recognition has reinforced the assumption that neuroinflammation influences, or even initiates AD pathogenesis, such that mutations increase the risk of AD in the gene responsible for the regulation of microglial activity. Cytokines substantially contribute to any aspect of neuroinflammation in AD. Increases in plasma and CNS levels of some cytokines substantially contribute to any aspect of neuroinflammation in AD [126]. Some cytokines that have increased plasma and CNS concentrations and the proinflammatory CNS microenvironment correlate with the likelihood of transformation to clinical AD from mild cognitive impairment. These results could explain why the absence of pathological changes, in AD or AD-related biomarkers, is associated with clinical AD if an embellished inflammatory response has been produced. In AD, microglia can bind via cell-surface receptors to soluble amyloid-β (Aβ) oligomers and Aβ fibrils [127]. The inhibition of receptors responsible for microglia swallowing or phagocytosis of Aβ fibrils appears to be a significant contributor to the pathology of AD, as it contributes to incomplete and inadequate removal of Aβ fibrils [128]. Local inflammatory factors are further triggered by continuous accumulation of Aβ caused by incompetent microglial-mediated clearance and chronic non-resolving inflammation is created as shown in Figure 2 [129].

Chemokines, also known as chemotactic cytokines, can control the migration of microglial to neuroinflammatory areas and spread into local inflammation in AD [129]. Proinflammatory cytokines such as TNF-alpha and IL-1β are often stimulated by chemokine release [130]. Upregulation of CCR3 and CCL2 chemokines and CCR5 receptor chemokines in reactive microglia have been recorded in patients with AD [131,132]. Other coexisting neurological disorders such as Alzheimer’s disease, Parkinson’s disease and exposure to neurotoxic stimulus cause damage to the BBB that along with tau and NFT aggregation, α synuclein and Aβ deposition and the presence of damage-associated molecular pattern (DAMP) and pathogen-associated molecular pattern molecules (PAMPs) result in NLRP3, TLR-4 and microglial activation, macrophage infiltration and nuclear translocation of NF-kB [133]. Activation of these signalling molecules induces oxidative and nitrative stress via increased lipid peroxidation, malonaldehyde (MDA), iNOS, peroxynitrite (ONOO) and causes neuroinflammation via increased levels of cyclooxygenase-2 (COX-2), leukotrienes (LTs), TNF-α, matrix metalloproteases (MMPs) as shown in Figure 2 [134]. Transient neuroinflammation involving resident glial activation and the production of several neuroinflammatory cytokines, including IL-1β, TNF alpha, and IL-6, may also occur. This neuroinflammatory cytokine induction may be induced as part of a coordinated interpretation of peripheral infection or insult in the CNS [135]. In AD, inflammatory cytokines such as TNF, IL-1, IL-12, and IL-23 might sustain microglial impairment. Proinflammatory factors such as IL-1, IL-6, and TNF-α have been linked to neurodegeneration in cerebral regions such as the hippocampus in vascular dementia. In Lewy body dementia, elevated expression of IL-1α and TNF-α has been found [136]. TNF-α and other cytokines were found to be higher in patients with frontotemporal dementia compared to controls [137,138].

## 7. Future Directions

The brain represents stasis from a cellular viewpoint, whereas the immune system depicts a dynamic motion. As the importance of neurodegenerative disease increases, these two perspectives merge. Nonetheless, recognizing and managing their interactions hold the key to a majority of late-set CNS diseases being avoided or delayed [139,140]. Microglia are activated in disease conditions, releasing proinflammatory cytokines which cause neuroinflammation and neuronal injury. Phagocytosis and autophagy are interrupted, contributing to the aggregation of proteins and mitochondrial disorders, leading to the enhanced generation of ROS that causes further inflammation [109,141,142]. While emerging proof indicates that inflammation performs a contributory role in the pathogenesis of AD, inflammatory markers have not yet been well identified as a valuable instrument for diagnosing AD. The discovery of inflammatory biomarkers to identify prodromal AD stages will be a significant part of future research [143,144,145].

Observational research encourages the use of non-steroidal anti-inflammatory drugs (NSAIDs) to protect from AD, while randomized controlled trials (RCTs) do not. To clearly understand NSAID use and AD risk, well-designed studies and creative approaches are needed. It is also critical to determine the best dose and duration of use for maximum gain and protection [146,147]. In chronic neurodegenerative disease, the ligand-receptor interactions in the CNS microenvironment, which maintains microglia under tight control in the healthy brain, are disrupted. However, the “when” and the “how” remain to be worked out. The concept of “activated microglia” is useful shorthand; however, it has hampered progress in understanding the richness of the microglia phenotype and the remarkable plasticity of these cells. The effect of chronic comorbidities and the resulting systemic inflammation and aging must be considered a major risk factor. The brain’s innate immune cells respond quickly to systemic events, and these responses are amplified in aging and in unhealthy brains. The limited tools that allow us to evaluate various states of in vivo microglia activation make it difficult to understand the involvement of neuroinflammation in the human CNS. This is a field with significant unmet needs; better PET ligands or other imaging modalities are critical. Recognizing the fact that manipulating the neuroinflammation is dependent on the immune system leads to the pathogenesis of chronic neurodegenerative disease and would provide several possible routes to delay the onset or progression [106,137].

## 8. Need for Guidelines

The involvement of various risk factors, from pathogenic to genetic, suggests that the occurrence and prevalence of dementia can be prevented through a public health approach, which can include an introduction of key measures that postpone or reduce cognitive deficit or dementia. The Global Action Plan on Dementia Response in Public Health 2017–2025 was adopted by the 70th World Health Assembly [18]. The action plan aspires to witness a society where dementia is avoided and patients who have dementia and their caregivers can live a good and healthy life. Besides getting support and care, they need to realize their capacity with equality and dignity. The plan of action covers seven areas of strategic interest and one of them is the reduction of dementia risk. The strategic management focuses on the WHO Secretariat developing, distributing, and spreading a base of facts to support policy measures to minimize modifiable dementia risk factors. That also includes having a database of evidence retrieved on the incidence of risk factors and the effects of their reduction and encouraging the formulation and carrying out of multi-sectoral interventions based on proof to reduce dementia risks. These guidelines are the first measures to help countries develop ways to delay or prevent the development of dementia [5,107,148].

## 9. Conclusions

Dementia is a global public health issue that is growing rapidly. It is clinically characterized by gradual cognitive decline with memory impairment, and can affect the level of cognition, interfering with social functioning in daily living activities, one of the biggest challenges of the 21st century. Dementia is responsible for huge economic losses for states, societies, individuals, and families. There are numerous primary care problems; dementia does not just progress along with age, but significantly influences caregivers’ mental health, healthcare providers, and the community at large.

**Figure 3:**
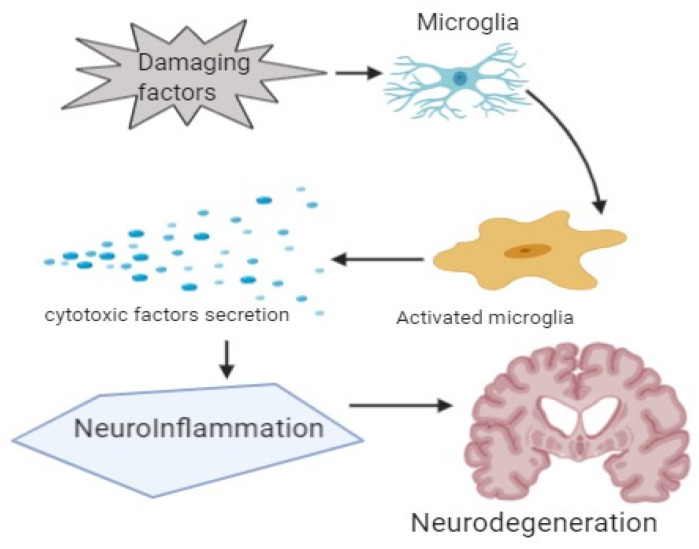
Showing graphical abstract.

In this review, the association of inflammation with dementia is discussed (Figure 3). In AD, deposition of the amyloid β-protein alone can cause an inflammatory response that leads to memory impairment and disease progression. Despite the chance that the deposition of the amyloid β-protein will precede “cognitive deficits” or “clinical manifestation” by decades, it can be speculated that endogenous or exogenous factors can alter the natural immunogenic response mounted by exposure of microglia to amyloid β-protein. Therefore, ecologically adjustable risk factors of AD, including obesity, traumatic brain damage, and systemic inflammation, can cause dementia through the continued neuroinflammatory drive.

## Figures and Tables

**Table 1:** Primary dementias as a clinical entity [8].

Risk Indicators	Cumulative Lesion	Neurodegeneration	Decline in Cognition
Non-modifiable	Extracellular amyloid proteins	Loss of synaptic	
Age Genetics	Tau (AD and FTD) intracellular protein	Neuroinflammation death in neurons	A decline in one or more memory visuospatial functions Language executive social cognition functions Complex attention
Modifiable			
Vascular risk, factor head injury, low education, poor hearing, depreciation, social isolation	Synuclein (LPD, PDD, MSA) PrPSC (prion disease) TDP-43(FTD) FUS(FTD)	Glial reaction	

**Table 2:** Studies using in vivo neuroinflammation imaging for human dementia patients.

S.NO	Neuroanatomical Areas with Substantially Greater Inflammation Compared to Controls in the Dementia Group	Other Findings	References
1	Inferior and middle temporal gyri, fusiform gyri, putamen, left amygdala, left posterior cingulate, left parahippocampal gyrus, inferior parietal lobes, and right pallidum in AD relative to controls. Inferior temporal gyri, fusiform gyri, and left parahippocampal in MCI compared with controls	Enhanced neuroinflammation in the left inferior temporal lobe differentiated AD relative to locomotion Regions with elevated inflammation exhibited the highest atrophy rate in AD for 12–24 months.	[97]
2	Lateral frontal cortices, prefrontal cortices, right mesial temporal cortex, left orbitofrontal cortex	Specific regional inflammation was linked with particular cognitive impairments. The task of image recognition and right superior frontal cortex, the role of orientation and left frontal lateral, left parietal cortex, and right superior frontal.	[98]
3	Temporal, frontal, parietal, cingulate cortices, and occipital cortices	Inflammation of cingulate gyrus posterior, parietal frontal cortices are associated with MMSE scores.	[99]
4	Striatum and cerebellum, anterior cingulate, dorsal parietal, lateral, temporal occipital cortices and medial prefrontal cortices.	No MMSE and inflammation association in any amount of interest.	[100]
5	Basal ganglia, substantia nigra, frontal lateral cortices. The lateral parietal, lateral temporal, cingulate, anterior and posterior cortices, medial occipital and lateral cortices in the occipital, temporal DLB poles, and precuneus compared to the controls	None reported	[101]
6	Sensorimotor cortices, prefrontal cortices, superior parietal, parietal cortices, middle, temporal, occipital cortices and posterior cingulate cortices in AD relative to controls. Inferior parietal lobules, middle and inferior temporal cortices, and precuneus had the greatest variations, but no difference was seen in thalamus, striatum, or cerebellum, white matter.	Age-related inflammation in the parietal cortex and striatum. Appearance in MCI and AD.	[102]

Mild cognitive impairment (MCI); Alzheimer’s disease (AD); mini-mental state examination (MMSE) and dementia with Lewy bodies (DLB).
